# Prevalence of Female and Male Vegan and Non-Vegan Endurance Runners and the Potential Associations of Diet Type and BMI with Performance—Results from the NURMI Study (Step 1)

**DOI:** 10.3390/nu14183803

**Published:** 2022-09-15

**Authors:** Katharina Wirnitzer, Derrick Tanous, Mohamad Motevalli, Gerold Wirnitzer, Claus Leitzmann, Renato Pichler, Thomas Rosemann, Beat Knechtle

**Affiliations:** 1 Department of Research and Development in Teacher Education, University College of Teacher Education, Tyrol, 6020 Innsbruck, Austria; 2 Department of Sport Science, University of Innsbruck, Tyrol, 6020 Innsbruck, Austria; 3 Research Center Medical Humanities, Leopold-Franzens University of Innsbruck, Tyrol, 6020 Innsbruck, Austria; 4 AdventureV & Change2V, 6135 Stans, Austria; 5 Institute of Nutrition, University of Gießen, 35390 Gießen, Germany; 6 Niederfeldstrasse 92, 8408 Winterthur, Switzerland; 7 Institute of Primary Care, University of Zurich, 8000 Zurich, Switzerland; 8 Medbase St. Gallen Am Vadianplatz, 9000 St. Gallen, Switzerland

**Keywords:** plant-based, diet, recreational, training, performance, running, half-marathon, marathon, ultra-marathon

## Abstract

This study aims to investigate vegetarian and mixed diet type prevalences among distance runners at running events around the world and associations with running-related patterns and performance. Following a cross-sectional approach, linear regression analyses were carried out to identify potential associations among body mass index (BMI), diet type, and average best performance times of half-marathon and marathon events for males and females. From a sample of 3835 runners who completed an online questionnaire, 2864 all-distance runners (age: 37 years; 57% females) were included in inferential analyses and categorized into dietary subgroups according to self-reports: 994 vegans (34.7%), 598 vegetarians (20.9%), and 1272 omnivores (44.4%). Significant associations were identified between kind of diet and best average time to finish (i) half-marathons in females where vegans (*p* = 0.001) took longer than omnivores, (ii) half-marathons in males where vegans (*p* < 0.001) and vegetarians (*p* = 0.002) took longer than omnivores, and (iii) marathons in males where vegans (*p* < 0.001) and vegetarians (*p* = 0.043) averaged slower than omnivores. Increased units of BMI (+1.0) in males influenced best runtimes: 2.75 (3.22–2.27) min slower for HM and 5.5 (5.69–4.31) min slower for M. The present study did not take detailed confounders into account such as runner motives or training behaviors; however, the results may provide valuable insight for running event organizers, nutrition experts, coaches, and trainers advising runners who adhere to a general diet type regarding the basic question of who participates in running events around the world.

## 1. Introduction

Distance running is a highly popular sport and healthy activity with millions of participants worldwide [1]. There are a variety of distance events (mainly 5 km, 10 km, half-marathon, marathon, and ultra-marathons) to take part in for accommodating every interest and physique [2]. Globally, a data report from the Strava 2021 Year in Sport, which included 95 million athletes, revealed that runners logged a total of 2.4 billion miles [3], and other reports have found that more than 100 million Europeans and US Americans combined are active runners [4,5]. Among the top five greatest marathon events on the global scale, the New York City Marathon ranks number 1, breaking the 50,000 benchmark with a total of more than 50,770 starters [6]. With such great interest, it is not surprising that the World Marathon Majors series includes six-city marathon events in Tokyo, Berlin, London, Boston, New York, and Chicago, comprising a total of more than 221,000 participants at the starting lines with over 180,000 completing all six events [7,8]. The massive participation and wide sociodemographic variety make distance running a particularly interesting area within endurance sports for scientific investigations [9].

According to the largest study of race results ever recorded up to 2018, running event participation has increased by 57% over the last decade [10]. In addition, the average finishing times have dramatically changed for females and males across race distances since 1986, even though groundbreaking technologies such as ‘carbon fiber plate shoes’ from sports companies may have led to the fastest marathon times ever recorded [10,11]. The slower average run times are likely due to a shift in the main motives of running participation in the direction of health (10%) and leisure (54%) instead of performance (36%) [12,13]. The most vital motive for running across sexes and all ages is the general health orientation and expectation of marked improvements to health [13,14], considering older runners at or above the age of 50 mainly follow running regimes for health maintenance and for preventing lifestyle diseases [15]. According to a recent survey conducted by ASICS, the tremendous impact of the COVID-19 pandemic in societies worldwide appears to have pushed the running motive shift even further toward the benefits for mental health [16].

As a natural low-intensive locomotion pattern, distance running requires, on the one hand, the appropriate fuel to perform best and most comfortably, and—as outrunning competitors is the goal of the sport—on the other hand, the motto, ‘higher, faster, further’ applies [13,17]. Nutrition is a basic variable to consider for every runner. Currently, the inconsistency of recommendations on what to eat for athletes and for health is overwhelming among major sports and nutritional bodies, such as the American College of Sports Medicine [18] or the British Dietetic Association [19]. However, in the world of running, reports of general diet type categories prevail among athletes, such as vegan (plant-based, excluding all animal products), vegetarian (partially plant-based, excluding animal flesh), or omnivorous diets (inclusive of plant and animal sources, including animal flesh, fish, dairy, egg, etc.) [20]. A current study that analyzed over 2 million Facebook posts across 132 countries found the sustainable, plant-based lifestyle interest as high as 18%, confirming the increasing trends forecasted previously by major analysts [21].

While the popularity of plant-based kind of diets is spreading faster than expected [22], anecdotal evidence among competitive athletes promoting vegan and vegetarian nutrition may be driving this accumulation [17]. Hence, vegan–vegetarian diets are currently booming in recreational and competitive sports [17]. Professional vegan endurance runners include Fiona Oakes (ultra-marathon running, set her fourth marathon world record in 2018) [23] and Scott Jurek (set multiple records, including the fastest Appalachian Trail completion of 2189 miles in 46 days, 8 h, 7 min in 2015) [24]. As cumulative evidence connecting the safety and benefits of plant-based diets for endurance sports arises, the consequence is that every social group or sports team will consist of at least one vegan person or athlete [17,25].

It is often believed among the public that when prepping for long-distance events, it is necessary to train with lengthy endurance runs the days and weeks before the planned running event to meet the high workload demands [26,27]. Inadequately increasing the training load may result in adverse health outcomes, including chronic pain and running-induced injuries [28], rather than enhancing performance and guaranteeing success [1]. Considering the physical and physiological differences between male and female endurance athletes [29], sex has been reported to be an indicator of training/racing behaviors and nutritional patterns of endurance athletes [30,31]. Specifically, evidence shows that sex-based differences in endurance performance may be influenced by race distance [32] and diet type [33]. Regardless of sex, consultation with a specialized professional in running could be highly beneficial for health, training adaptations, and performance in runners [34,35]. In this regard, it has been reported that endurance athletes of lengthy distances, marathoners and ultra-marathoners, for example, are more likely to consult with performance specialists such as sport scientists and sports medicine doctors [36].

A recent study found around 10% prevalence of vegan or vegetarian diets among marathoners [37]. While there is an expanding prevalence of plant-based diets, notably among endurance runners, there are only limited data considering this topic [38,39]. Given the background numbers, plant-based athletes are no longer a fringe group, and thus, the importance of providing data to overcome the lack of information as well as practical knowledge for expanding individualized training and nutritional strategies appears to be crucial to examine the associations between diet type and running-related characteristics in a large sample of vegan and non-vegan endurance runners. To date, there have been several studies conducted on endurance performance linked to vegan/vegetarian diets [12,25,40,41,42,43,44], but the results are yet to be conclusive and have found little to no differences. Moreover, investigations have also studied the training habits of distance runners among various race lengths, whether distinctly [45,46] or comparatively [36,40]. However, no study has, to date, examined the prevalence of different diet types among distance runners attending running events worldwide and their associations with running performance concerning women and men separately. Therefore, the present study aimed to investigate the prevalence, sociodemographic, and anthropometric comparisons of endurance runners from anywhere in the world and identify potential relationships with racing performance among females and males following vegan and non-vegan diets based on a large sample. The present investigation hypothesizes that there is a difference in best time racing performance at half-marathon and marathon recreational events based on diet type.

## 2. Materials and Methods

With a cross-sectional design, The Nutrition and Running High Mileage (NURMI) Study was arranged in three steps. It was created as a follow-up to a previous study on vegan ultra-endurance mountain biking over eight consecutive stages [47,48] and is considered the largest study of running in Europe (www.nurmi-study.com/en, accessed on 14 September 2022). The methodology of NURMI Study Step 1 was described in detail (ethics approval, participant recruitment, etc.) elsewhere [49]; in short, as preliminary study using a cross-sectional approach, Step 1 aimed to evaluate the prevalence and basic characteristics of running and racing behavior of vegan and non-vegan recreational runners who are active in events (all distances, all levels from recreational to elite). The interested reader is referred to all NURMI Step 1 publications for further details [36,38,40].

A short online survey offered in English and German within the NURMI Study Step 1 had to be completed by participants. Although the core regions for study sample recruitment were European countries, the NURMI Study’s information, including an online survey, was spread across the globe for the international runner community. The survey was introduced with a written procedural description, and informed consent of participants was required to take part in the study. Afterwards, they completed the survey, which included basic and complementary questions and controlled for diet type and running activity. Basic questions were on sociodemographic attributes, current adherence to a specific diet type (with a minimum adherence of at least six months), and distances being active in running (training, races).

Along with the basic questions for the classification of participants, including (i) adherence to current diet (mixed, vegetarian, vegan) along with its duration of adherence and (ii) the preferred running distance for events, runners were asked to provide complementary data about their sociodemographic and anthropometric characteristics, food intake of specific items, dietary (inclusive fluid) intake on race days, weekly time spent in running training), period of time to prepare for the main running event, aim of partaking in a running race (performance vs. enjoyment approach), preparation strategies for competition, event participation over several distances (<21 km, half-/full-/ultra-marathon), number of successfully completed specific distances, and individual best runtime over the respective distances. All basic and complementary data were collected based on a self-report approach using online questionnaires. Diet quality and personal running motivations were not included as a part of this investigation.

For successful study participation, five inclusion criteria were initially required to be fulfilled in order to be included in the final sample: written informed consent (1), minimal age of 18 years (2), Step 1 questionnaire completed (3), participating and completing at least one running event over 5 km distance in the past two years (4), and being active in running-related physical activities associated with the self-reported race distance (5). Participants meeting all inclusion criteria were registered in the study to avoid a permanent loss of valuable data, which aided in a larger sample and increased the data representation provided for the current results.

For exclusion criteria, the BMI-associated approach based on the WHO [50,51] was implemented to regulate for a minimum status of health and a minimal level of fitness and further aid in enhancing the reliability of data sets. According to the WHO [50,51], increased health risk results from a body mass index (BMI) higher than BMI_NORM_ (range: 18.50–24.90 kg/m^2^; that corresponds to achieving an optimum state of health) is specified for co-morbidities at a BMI of 25.0–29.9 kg/m^2^ and moderate-to-severe co-morbidities at a BMI > 30 kg/m^2^ [50,51]. Other health protective strategies besides running are required for people with a BMI ≥ 30 to safely reduce body weight (BW) first [43]. Therefore, the calculated BMI (BMI_CALC_) was classified into three categories (kg/m^2^): ≤18.49 < BMI_NORM_ ≤ 25, and participants with a BMI ≥ 30 were excluded. Additionally, as a second exclusion criterion for the present investigation, runners must have reported their best time for completing a half-marathon or marathon.

Dietary subgroup classification of participants was based on the following groups: omnivorous (also known as the Western diet, includes no dietary restriction), vegetarian (no consumption of meat or fish), or vegan (no consumption of products from animal origin: meat, processed meat, fish, seafood, shellfish, milk, dairy products, eggs, or honey) [20]. Participants must have followed their respective diet for the minimum duration of 6 months to be included in the omnivore, vegetarian, or vegan subgroup. Moreover, participants were initially categorized into three subgroups regarding race distances: half-marathon (HM), marathon (M), and ultra-marathon (UM, distances longer than a marathon). The minimum ultra-marathon race completed was 50 km, and the longest race was 160 km. To regulate for HM or M race completion, the participants best HM or M times were checked and confirmed by random sample selection. In addition, 622 highly motivated runners that had not successfully finished at least a half-marathon race before but instead competed in shorter distances (<21 km, mainly 5 km, 8 km, and 10 km) provided accurate and high-quality data. To avoid a permanent data loss of the shorter than half-marathon distance runners, those who met all inclusion criteria were pooled together as an additional race distance subgroup.

Statistical analysis was performed by the statistical software R version 4.1.1 (2021-08-10), Core Team 2021 (R Foundation for Statistical Computing, Vienna, Austria). The descriptive statistics were summarized using means and standard deviations (SD) as well as medians and interquartile ranges (IQR).

Univariate analysis was used to describe the distribution of recreational runners considering diet type and was conducted with the Chi-square test (χ^2^; nominal items) and Kruskal–Wallis test (ordinal and metric items) to examine the association between dietary subgroups (F distribution was used for approximation). Multiple linear regression analyses were used to examine significant differences in performance of races (best time over the half-marathon and marathon distances) and were stratified by sex, while BMI was considered the potential confounder. Inspection of the graphs of predicted vs. residual and normal Q-Q residual plot was performed to verify the assumptions of the regression analysis. Dietary subgroup-related differences by race distance of runners are shown with effect plots stratified by sex (confidence interval set at 95% (95%-CI)); predictor effects and 95%-CI were calculated with the R effects package.

The statistical significance level was set at *p* ≤ 0.05.

## 3. Results

**Figure 1:**
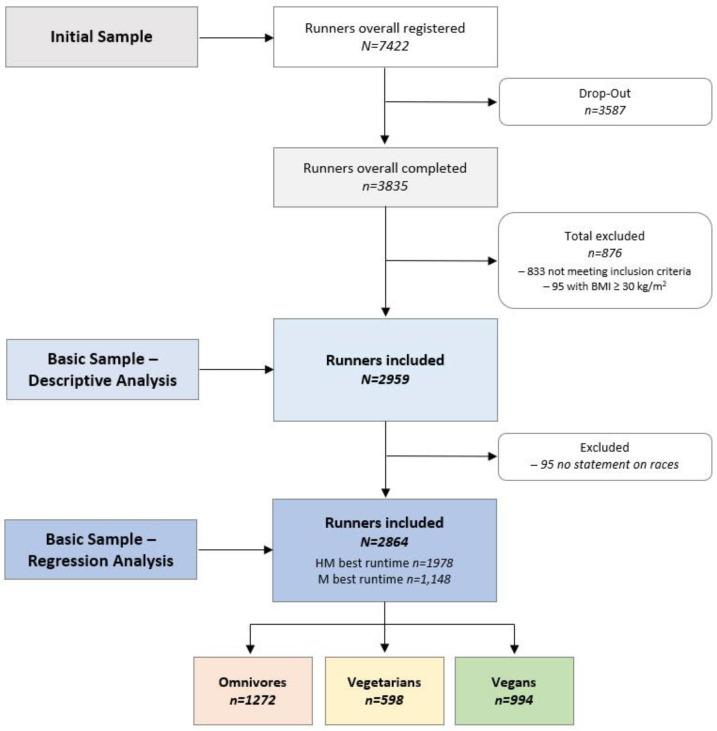
Participants’ enrollment and classification based on dietary subgroups. Omnivores—do not impose any restrictions on themselves with regard to the origin of source of food. Vegetarians—consume egg and/or dairy products but no flesh foods at all. Vegans—consume no foods and ingredients from animal origin.

A total of 3835 runners submitted the survey with complete statements and a returning response rate of 52% of all that started filling in the online survey. Of these, 43 participants were excluded due to inconsistent or conflicting data sets, and 833 participants did not meet the basic inclusion criteria, 95 of whom were excluded due to reporting a BMI of ≥ 30. Ultimately, 2959 all-distance runners were considered the sample for descriptive analysis, and 95 runners were excluded before regression analysis due to the lack of information on running races and/or half-marathon and marathon best times. The final sample of endurance runners in the statistical analysis included 2864 fit participants (57% female) from around the world, including Europeans (*n* = 2789) primarily but also a total of 75 non-Europeans: North and South Americans (*n* = 70), Asians (*n* = 4), and one answer was not specified. Figure 1 shows the flow of participants’ enrollment for the NURMI Study Step 1.

The participants had a median age of 37 years (range 18–74), and 83% had a BMI within the norm (*n* = 2394), while 5% and 12% were underweight (*n* = 138) and overweight (*n* = 332), respectively. Regarding diet type, 2746 recreational runners (96%) correctly self-reported their diet type, and 118 participants (4% overall) were relocated to the appropriate diet type based on control questions: 7 self-reported vegan runners (4 to omnivores and 3 to vegetarian samples), as well as 111 self-reported vegetarians (all to the omnivores subsample). Thus, 35% of the participants followed the vegan diet (*n* = 994), 21% of the participants followed the vegetarian diet (*n* = 598), and 44% of the participants followed the omnivorous diet (*n* = 1272). According to the participants race distance classification, 22% were <21 km runners (*n* = 622), 36% were half-marathoners (*n* = 1032), and 42% were marathoners or ultra-marathoners (*n* = 1210). For training parameters, runners reported a running frequency (e.g., number of days with running training sessions) of 3 days/week (range: 1–14 sessions), a weekly average of 43.3 (±7.3, range: 5–219.5) kilometers, and an average weekly duration of 4.7 (±2.8, range: 0.6–24) hours. Some participants (*n* = 96) completed a performance assessment as part of their training and race preparation. The most common professional advice sought by participants was from a trainer (*n* = 273), which was followed by a sport scientist (*n* = 35) and a sports medicine doctor (*n* = 29). Before participating in a running event, 73% of the runners (*n* = 2103) trained for 1–4 months, 22% (*n* = 624) trained for 4–8 months, and 5% (*n* = 137) trained for nine months or more.

The runners reported their nutrient and liquid intake on competition day with the highest response of adopting a different intake for competition day (*n* = 1119), which was followed by a dietary intake that is the same for training days (*n* = 917), eating whatever the individual feels like (*n* = 604), and the same intake as rest days or always (*n* = 224). Participants reported the number of their completed events for each race distance; a frequency of 1–2 completed events was most common across all distances (HM: *n* = 762; M: *n* = 513; UM: *n* = 131), although many participants had completed between three and seven events of a specific distance (HM: *n* = 730; M: *n* = 382; UM: *n* = 111), and some had completed more than seven events of a specific distance (HM: *n* = 482; M: *n* = 248; UM: *n* = 40).

Significant between-group differences (Table 1) were observed for (i) sex (*p* < 0.001), with most females following vegan or vegetarian diets and most males following an omnivorous diet; (ii) age (*p* < 0.001), with the vegans being the youngest and omnivores being the oldest; (iii) BW (*p* < 0.001), in which vegetarians had the lowest median BW (IQR 63, range: 40–98) and omnivores the highest (IQR 69, range: 40–105); (iv) BMI category (*p* < 0.001), where vegetarians (87%), followed by vegans (85%) had the highest prevalence of BMI norm, the vegans were most prevalent (7%) among the underweight, and omnivores were the most prevalent (16%) among the overweight participants; and (v) race distance (*p* < 0.001) where the most prevalent diet among the <21 km runners was the vegan diet (41%), whereas the omnivorous diet was most prevalent among half-marathon runners (42%) as well as (ultra-)marathoners (50%).

In Table 2, the best times on average to complete half-, full, and ultra-marathon events are displayed by male and female runners across dietary subgroups.

**Figure 2:**
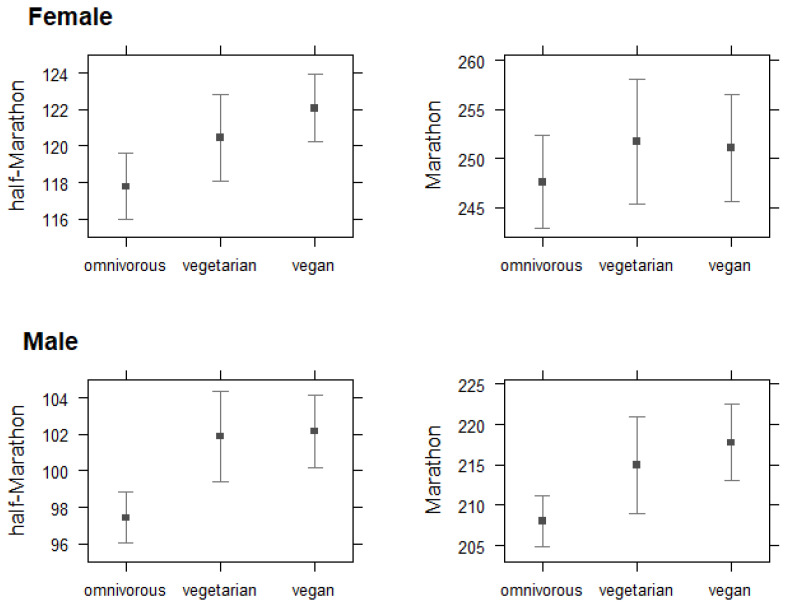
Interactions between dietary subgroups (in *x*-axis) and best runtime in minutes. Effect plots with 95%-CI across race distances (half-marathon (HM) and marathon (M)) and sexes (females and males). HM distance finishers with best runtime (females: *n* = 1051; males: *n* = 927). M distance finishers with best runtime (females: *n* = 486; males: *n* = 662). *Note.* The 95% confidence intervals (CIs) and *p*-values were computed using the Wald approximation.

We fitted a linear model (explaining 12% of the variability of HM and 11% of M distance for BMI, and 11% of the variability of HM and 14% of M distance for diet type; *p* < 0.001) to predict the marginal effect of BMI and kind of diet on female and male runner performance of HM and M distance by average best runtime (minutes). Figure 2 displays the sex-specific performance of best times for HM and M distances on average by dietary subgroups. Significant relationships were found between sex and diet type in male and female runners’ best runtime on average at completing the HM and M distance.

Compared to the main effect for best runtimes in male omnivores over the HM distance (34.1 min (95%-CI: 45.3, 22.9)), we found vegans to take 4.76 min (95%-CI: 7.25, 2.27; *p* < 0.001) and vegetarians 4.48 min (95%-CI: 7.35, 1.61; *p* = 0.002) longer to finish an HM on average; over the M distance, male vegans were found to take 9.76 min (95%-CI: 15.5, 3.98; *p* < 0.001) and vegetarians 6.99 min (95%-CI: 13.8, 0.21; *p* = 0.043) longer to finish an M on average versus male omnivore marathon runners (81.6 min (95%-CI: 45.3–22.9). Omnivore females on average finished both the HM (56.8 min (95%-CI: 67.7, 45.9); *p* < 0.001) and M (108.0 min (95%-CI: 138.0, 77.4); *p* < 0.001) distance significantly faster than vegan women at HM events (4.31 min (95%-CI: 6.91, 1.72); *p* = 0.001), but not vegetarian female half-marathoners (2.69 min (95%-CI: 5.68, −0.3); *p* = 0.078), and also not over M distance (3.5 min (95%-CI: 10.8, −3.76); *p* = 0.344), while female vegetarian marathoners finished slowest (4.14 min (95%-CI: 12.1, −3.78); *p* = 0.305).

## 4. Discussion

The NURMI Study was developed to investigate the differences between recreational runners with a specific focus on diet type by including a large sample and respectable dataset based on the premise that scientific evidence on endurance athletes adhering to plant-based (vegan, vegetarian) diets is lacking. The NURMI Study (Step 1) aimed to answer the free form question, “who starts at running events?”. However, this investigation aimed to analyze the vegan, vegetarian, and omnivorous dietary prevalences among recreational runners at the start of races across the world and analyze the participants’ sociodemographic, anthropometric, and performance characteristics over half-marathon and marathon distances. The present investigation is the first article to distinguish the best running performances of vegan versus non-vegan female or male participants on average, including the prevalence of vegan, vegetarian, and omnivorous runners at the starting line of running events worldwide, and it verifies the hypothesis that there is a difference in recreational HM and M best time performance based on diet type.

As the main findings, significant associations were found between (i) sex and diet type prevalence with more females following vegan or vegetarian diets; (ii) age and diet type with vegans being youngest and omnivores being oldest; (iii) BW and diet type with vegetarians having the lowest BW and omnivores having the highest; (iv) BMI and diet type with vegetarians having the highest prevalence of BMI_NORM_, vegans were the most prevalent among the underweight, and omnivores were the most prevalent among the overweight participants; while (v) females reporting omnivore diet were significantly faster on average to complete an HM event compared to vegans; and (vi) males reporting omnivore diet were significantly faster to complete HM and M events compared to both vegans and vegetarians.

Across the scientific literature on nutrition in runners, comparisons of general diet types of endurance runners remain largely unstudied. Considering the well-established fact that the non-medical approach of being physically active over the lifespan is the key to long-term health through the maintenance of physiological systems [52], it is not surprising that the health status [12,43,44] and the quality of life of recreational endurance runners is high [42]. However, it is interesting that endurance runners do face particular nutrition-related health risks, which may arise from a low dietary energy intake compared to the higher exercise-induced energy demand of regular distance running [53], especially in females [54]. In addition, any particular diet type may also play a role in a person’s nutrient status. While the vegan/vegetarian population is often criticized for lacking the so-called critical nutrients of protein, vitamin B12, vitamin D, iron, calcium, or essential fatty acids [55], the American Academy for Nutrition and Dietetics has maintained their position for over 40 years regarding their approval for such diets, which they advocate for being nutritionally adequate, healthful, and may help to prevent non-communicable diseases over the lifespan (i.e., heart diseases, type II diabetes) when planned appropriately [20,56]. The omnivore diet, on the other hand, is consistently found to be associated with higher incidences of overweight/obesity [57,58] and may also pose specific nutrient concerns, including insufficient vitamins B12 and D for the general population and endurance runners alike [17,59]. However, a well-designed omnivorous diet containing predominantly whole plant-based foods, lean meat (fish, chicken, turkey, or red meat), and skim milk may support health in the short term as well [60]. Additionally, the patterns of supplement intake in endurance runners may be unrelated to any particular diet type but instead support the notion of health and lessen the nutritional deficiency concerns among endurance runners [61]. Both diet type and dietary supplements, however, are considered nutritional strategies that enhance performance, accelerate recovery, and prevent or reduce gastro-intestinal discomfort, and they can be used by recreational athletes to cope with the additional nutritional requirements of regular exercise with little to no adverse consequences [17,31,61]. For more detailed information on dietary supplementation of recreational endurance runners, the interested reader is referred to previous NURMI publications [31,61], including dietary subgroups specifically [62]. While endurance runners are often hailed as being healthy for their exercise habits [63], it is interesting to find by the present study that more than one-fifth of the participants (*n* = 604) showed little to no concern with their nutrient and liquid intake, especially for competition day regarding the questionnaire response, “I eat and drink what I feel like.” Considering that vegans/vegetarians typically adhere to their diets for a specific purpose, whether health, environmental, ethics/animal welfare, or a combination of concerns [64], it is unlikely that these participants would report eating whatever they would like to. Given the evidence of previous publications from the NURMI lab, it has been reported that regularly active vegans have a higher level of health consciousness, and therefore, most beneficially uphold their health status by paying particularly close attention to the foods they consume [43].

In the study at hand, there is a higher proportion of female vegan/vegetarian participants, and the males more frequently reported an omnivorous diet, which is consistent with general populations [57,58,65]. In addition, the vegans, followed by the vegetarians, were found to be younger than the participants reporting the omnivore diet, which may be related to the veggie boom across younger generations [17,21,22]. Regardless of sex and age, the total prevalence of vegan and vegetarian diets appears to be rising worldwide [17,21,22,65]. Similar to general populations and in line with the findings from the present study, an increasing trend in the prevalence of plant-based diets has been reported in distance runners [66] even with a higher growth rate than general populations [37,66]. Evidence shows that not only the popularity of vegan/vegetarian diet adherence was greater among ultra-marathoners than endurance runners in shorter distances, but they also reported longer adherence to their diets [66]. It has also been reported that the prevalence of vegan/vegetarian diets is associated with sociodemographic parameters in endurance runners, i.e., educational background, sex, BMI, status of health [40,43,66], and general populations, i.e., sex, ethnicity, and educational background [39,67]. The COVID-19 pandemic has been found to be associated with an increase in the prevalence of vegan/vegetarian diets [68]. Table 3 represents the worldwide percentages of vegan/vegetarian diets and differentiated details for specific continents [69].

However, the total worldwide prevalence includes all nations, such as developing countries, which may not be comparable to more developed nations. Asia shows the highest proportion of vegan diet prevalence (3%), and South America has the highest vegetarian prevalence (12%), which may be due to the fact that the veggie lifestyle is viewed as healthy and substantially more environmentally sustainable [69,70].

In addition to differences in the prevalence of females and males among the dietary subgroups, differences were found between BW and dietary subgroups; vegetarians, followed by vegans, had the lowest BW, which may be related to the fact that more vegetarians/vegans were female, and therefore, it is not uncommon to see a lower BW in comparison to a group with more males [71]. However, a greater proportion of the vegetarian, and even the vegan, subgroup was within the BMINORM category compared to the omnivores. In addition, the vegans were the most prevalent among the underweight participants (*n* = 68, 7% of vegans), and the omnivores were the most prevalent among the overweight (*n* = 206, 16% of omnivores). Although age was not used as a control for this finding, the strength in the direction of effect regarding overweight omnivores may even be reduced based upon the exclusion criteria of obese participants with a BMI > 30 kg/m. Sex-specific associations showed that the males with higher BMI in this study finished slower at HM and M distances. With every increasing unit of BMI (+1.0) in males, the best runtime is 2.75 (3.22–2.27) min slower for HM and 5.5 (5.69–4.31) min slower for M. Likewise, the finding of a greater underweight prevalence among vegan/vegetarian endurance athletes is a heightened concern for these particular diets [53,54]. Consistent with this finding, it has been reported that vegetarian and vegan diets could be considered effective strategies to reduce overweight and obesity, and on the other hand, the omnivorous diet has been found to have less favorable outcomes in weight reduction purposes [65,72]. While this result is in line with other studies [57], it is advisable for vegans/vegetarians, especially endurance athletes such as distance runners, to take more consideration when planning their day-to-day calorie consumption in order to achieve energy balance and meet the high energy demands of active endurance running [17]. For omnivore endurance athletes, it appears more advisable to promote greater consumption of the proportion of whole plant-based foods to animal products with their day-to-day diets, while whole plant-foods are nutrient-dense and at the same time contain low energy-density and provide considerable satiety through high amounts of fiber [59], which is ideal for sustaining a healthy body weight [73]. From a performance viewpoint, however, the optimal BMI for the best performance of distance running was found to be 19–20 kg/m^2^ [43,74].

For centuries, it has been considered that there is a connection between an athlete’s diet and their performance [75]. Considering the marginal effects of diet type, compared with runners on a mixed diet, we identified an increase in Time-Average over the HM race distance by males following the vegetarian diet (4.48 min) and males following the vegan diet (4.76 min), also with male vegans taking significantly longer to finish a marathon (9.76 min) but not male vegetarians (6.99 min). These results are, however, inconsistent with a study on laboratory physical exercise tests among omnivore, vegetarian, and vegan recreational runners, which found no difference between performance-related parameters in males, although this study included only 76 participants (29 of which were males) and did not report performing a power analysis to determine the minimum sample needed to detect statistical significance [41]. Interactions from females only found vegan HM runners to take significantly more time to complete, 4.31 min, but not for vegetarians or marathoners. While the result for marathoners is consistent with another recent study that found no difference in female runner performance based on diet type, the HM event result is inconsistent with other findings suggesting no difference [41]. Therefore and generally, considering the wide variety of performance contributors from individual training behaviors (total duration, frequency, intensity, type, etc.), running/racing experience, and especially personal motives to race day environmental conditions (season, temperature, altitude, incline/decline mileage, etc.) and personal strategies (supplementation, use of performance-enhancing substances, etc.) [1,13,36], interpretation of the best time performance results should be with caution. A lack of comparable studies distinguishing vegans from vegetarian participants limits further interpretation regarding endurance running performance, particularly individual best records.

As with every investigation, some limitations need to be addressed. The NURMI Study was established as a cross-sectional design with a questionnaire-based approach of self-reporting, and therefore, over- or under-reporting may have impacted the reliability of the data. Correspondingly, the implementation of control questions throughout different sections of the questionnaire minimized the likeliness of discrepant reports, especially for diet and race distance. A randomized selection of participants based on diet type was not conducted as the main aim of the NURMI Study to assess as many participants as possible. However, the major strength of the present investigation and the NURMI Study (especially Step 1) is the inclusion of a large sample of participants aiding in the representativity of the findings. Another limitation that may have affected the present results is the greater proportion of vegans (35%) and vegetarians (21%) compared with worldwide (9%) and continental estimates (9–14%) despite the fact that not all active vegan/vegetarian runners were within reach. However, this occurrence can be partially clarified by participant pre-selection, which may have had an effect on the results. In this regard, in spite of the lack of evidence on vegans and vegetarians explicitly, the fact that vegans/vegetarians might be interested in participating in such lifestyle-related investigations may not only partially justify the higher prevalence of vegan and vegetarian diet but also may affect the representativeness of other findings. Specific seasons could also be related to the numbers of vegan/vegetarian diets in self-reports, where flexible vegans and vegetarians, or “flexitarians”, may give different reports about their kind of diet in January compared to December (the time when many people make celebration-related dietary exceptions) [76]. Regarding the best time race performance results on diet type, several confounders were not taken into account that may likely influence the findings, such as running and racing experience and history, personal running motives, training behaviors, race conditions, or even individual diet quality and further nutritional characteristics. Likewise, the unequal distribution of participants based on diet type and racing distance was not controlled for, although runners of greater distances are known to perform better on average at long-distance events than shorter distance runners [36]. Thus, as the greatest proportion of <21 km runners was vegan, this may have limited the effect size of their best time performance compared with omnivores at half-marathon and marathon events. However, the main NURMI Study (linking Step 2 with Step 3) was designed to include detailed analyses with a large population comparing omnivorous, vegetarian, and vegan recreational athletes on running performances; thus, future publications with important confounders of running performance are planned. Correspondingly, another major strength of the present investigation is the exploratory approach, which was designed to generate hypotheses for future research considering the missing gap of diet type, especially vegan versus non-vegan, and performance comparisons of recreational endurance runners. It was anticipated that most of the participants would be Europeans (97%), as the main target population of the NURMI study was Germany, Austria, and Switzerland (D-A-CH countries); however, the determination of this study population (recreational endurance runners especially) may have increased the accuracy of their reports, which also led to the inclusion of non-European natives and contributed to an improvement in the quality of the data generated. The inconsistency of dietary proportions in our sample compared to general populations might be a result of several factors: (i) Germany, the largest German-speaking country, with a population of 82 million, has a large vegetarian and vegan population [77]; (ii) there appears to be a higher proportion of vegan/vegetarians among distance runners, which is consistent among other studies as well [17,37,40,66]; (iii) the sex differences in health consciousness, as females (who were significantly more than males in the present study) are reported to be more interested in following some kind of plant-based and healthy diet [33,43,78,79]; and (iv) vegans may have been more apt to participate given the lack of studies comparing their athletic performances distinctly [41]. In light of this study’s limitations, however, valuable drawbacks may be present in terms of who starts at running events, especially since the NURMI Study is the first study including a large sample with a distinct focus on vegan and vegetarian runners, which is of great importance for running event organizers but also for experts of nutrition such as sport nutritionists and dietitians as well as running coaches and trainers that advise athletes who adhere to some general diet type. In addition, the present findings may contribute to future studies in specifying whether performance differences exist in a homogeneous sample of endurance runners following vegan, vegetarian, and omnivorous diets by addressing more confounding variables.

## 5. Conclusions

The rise in the popularity of distance running alongside the expanding percentages of endurance athletes following vegan/vegetarian diets are two recent phenomena during the past decades that require joint attention by scientific investigations. In the present study, the percentages of the vegan and vegetarian diet among endurance runners was greater than the numbers of general populations, which is consistent with the findings of previous reports. Present findings also identified some potential associations between kind of diet and distance running best time performances in both males and females, suggesting the importance of dietary classifications in running- and performance-related variables. Despite the differing sociodemographic and anthropometric characteristics based on diet types, the present results are conclusive in showing that vegan and vegetarian diets seem to be appropriate for participating in distance running activities, particularly at the recreational level; however, increasing day-to-day calorie consumption is advisable for underweight runners. More detailed experimental research is needed to explicate the association between diet type and performance, as there were running differences identified among vegans, vegetarians, and omnivores in self-reported individual records. Findings from this investigation may provide beneficial evidence, which may be insightful for sport scientists, trainers, coaches, and nutrition specialists when advising training and/or nutritional strategies for vegan and vegetarian distance runners. Therefore, future research, including experimental approaches, should be designed to further examine the differences in health and performance among distance runners following different diet types across various sociodemographic strata.

## Figures and Tables

**Table 1:** Anthropometric Measurements and Demographics of Participants Displayed by Dietary Subgroup.

	Total *n* = 2864 (100%)	Vegan *n* = 994 (35%)	Vegetarian *n* = 598 (21%)	Omnivore *n* = 1272 (44%)	Statistics
**World Region**					
European	2789 (97%)	940 (95%)	592 (99%)	1257 (99%)	
Non-European	75 (3%)	54 (5%)	6 (1%)	15 (1%)	
**Sex**					χ^2^_(2)_ = 87.58; *p* < 0.001
Females	1628 (57%)	647 (65%)	381 (64%)	600 (47%)	
Males	1236 (43%)	347 (35%)	217 (36%)	672 (53%)	
**Age**	37	34	35	40	F_(2, 2861)_ = 44.05; *p* < 0.001
(years; median)	(IQR 17; 18–74)	(IQR 15; 18–70)	(IQR 17; 18–73)	(IQR 17; 18–74)	
**Body Weight**	66	64	63	69	F_(2, 2861)_ = 50.89; *p* < 0.001
(kg; median)	(IQR 16)	(IQR 15)	(IQR 14)	(IQR 16)	
**Height**	1.73	1.72	1.72	1.74	F_(2, 2861)_ = 14.71; *p* < 0.001
(m; median)	(IQR 0.13)	(IQR 0.14)	(IQR 0.13)	(IQR 0.12)	
**BMI**_CALC_ (kg/m^2^; median)					χ^2^ _(4)_ = 69.45; *p* < 0.001
BMI_NORM_	2394 (83%)	842 (85%)	518 (87%)	1034 (81%)	
<BMI_NORM_	138 (5%)	68 (7%)	38 (6%)	32 (3%)	
>BMI_NORM_	332 (12%)	84 (8%)	42 (7%)	206 (16%)	
**Race Distance**					χ^2^_(4)_ = 43.51; *p* < 0.001
<21 km	622 (22%)	257 (26%)	142 (24%)	223 (18%)	
HM	1032 (36%)	382 (38%)	215 (36%)	435 (34%)	
M/UM	1210 (42%)	355 (36%)	241 (40%)	614 (48%)	

Note: Results are shown as total numbers, percentage (%), and median (IQR) with range (min–max). BMI_CALC_—Body Mass Index calculated and categorized following the WHO [50,51]. BMI_NORM_ = 18.50–24.99; <BMI_NORM_ < 18.50; >BMI_NORM_ ≥ 25.00. <21 km—less than half-marathon (mainly 5 km, 8 km, and 10 km). HM—half-marathon. M/UM—marathon/ultra-marathon. *p*—*p*-value for difference among groups. χ^2^ statistic calculated by Pearson’s Chi-squared test and F statistic calculated by Kruskal–Wallis test. Vegans—consume no foods and ingredients of animal origin. Vegetarians—consume egg and/or dairy products but no flesh foods at all. Omnivores—do not impose any restrictions on themselves with regard to the origin or source of food.

**Table 2:** Best average runtimes (minutes) of female and male runners displayed by dietary subgroup.

***Females***	**Vegan**	**Vegetarian**	**Omnivore**
***(N = 1628)***	**(*n* = 647)**	**(*n* = 381)**	**(*n* = 600)**
HM	122 ± 20.4	119.7 ± 19.4	118.3 ± 19.6
M	251.3 ± 42.6	249.3 ± 36.1	248.8 ± 35
UM	791.9 ± 213.3	779.7 ± 223.5	778.4 ± 146.8
***Males***	**Vegan**	**Vegetarian**	**Omnivore**
***(N = 1236)***	**(*n* = 347)**	**(*n* = 217)**	**(*n* = 672)**
HM	100.3 ± 17.7	100.7 ± 16.5	98.7 ± 17
M	213.6 ± 32	212.9 ± 33.3	210.5 ± 33.5
UM	742.9 ± 212.3	759.9 ± 162.4	725.1 ± 204

Note: Data are presented as mean ± standard deviation. HM—half-marathon; M—marathon; UM—ultra-marathon. Vegans—consume no foods and ingredients of animal origin. Vegetarians—consume egg and/or dairy products but no flesh foods at all. Omnivores—do not impose any restrictions on themselves with regard to the origin or source of food.

**Table 3:** Worldwide percentages of vegan/vegetarian diets [69].

		Vegan	Vegetarian
** Total Worldwide**	2020	2%	7%
** Pacific Europe**	2020	2%	7%
** North America**	2020	2%	9%
** South America**	2020	2%	12%
** Africa/Middle East**	2020	2%	8%
** Asia**	2020	3%	8%

## Data Availability

The data sets generated during and/or analyzed during the current study are not publicly available but may be made available upon reasonable request. Subjects will receive a brief summary of the results of the NURMI Study if desired.

## References

[1] Boullosa D., Esteve-Lanao J., Casado A., Peyré-Tartaruga L.A., Gomes da Rosa R., Del Coso J. (2020). Factors affecting training and physical performance in recreational endurance runners. Sports.

[2] Shipway R., Holloway I. (2010). Running free: Embracing a healthy lifestyle through distance running. Perspect. Public Health.

[3] Strava Press Strava 2021 Year in Sport. San Francisco/USA. https://1n4rcn88bk4ziht713dla5ubwpengine.netdna-ssl.com/wp-content/uploads/2021/12/YIS-2021-Press-Book-United-States.pdf.

[4] Scheerder J., Breedveld K., Borgers J. (2015). Running Across Europe: The Rise and Size of One of the Largest Sport Markets.

[5] Sports & Fitness Industry Association Running/Jogging Participation Report 2020. https://www.sfia.org/reports/844_Running-Jogging-Participation-Report-2020.

[6] Top 5 Biggest Marathon Events Worldwide: STATISTA (2018) Größte Marathonläufe Weltweit Nach Anzahl der Finisher im Jahr 2018. https://de.statista.com/statistik/daten/studie/380316/umfrage/marathonlaeufe-weltweit-nach-anzahl-der-finisher/.

[7] The Abbot World Marathon Majors The Pursuit of Greater Potential: Six Cities, Three Continents, One Remarkable Goal. https://www.abbott.com/marathon.html#:~:text=More%20than%20180%2C000%20runners%20will,TCS%20New%20York%20City%20Marathon.

[8] Abbott World Marathon Majors Abbott World Marathon Majors. Welcome to Abbott World Marathon Majors: Where Champions Run. https://www.worldmarathonmajors.com/.

[9] Reusser M., Sousa C., Villiger E., Cruz J., Hill L., Rosemann T., Nikolaidis P., Knechtle B. (2021). Increased participation and decreased performance in recreational master athletes in “Berlin Marathon” 1974–2019. Front. Physiol..

[10] International Association of Athletics Federations The State of Running. https://runrepeat.com/state-of-running.

[11] Muniz-Pardos B., Sutehall S., Angeloudis K., Guppy F., Bosch A., Pitsiladis Y. (2021). Recent Improvements in Marathon Run Times Are Likely Technological, Not Physiological. Sports Med..

[12] Wirnitzer K., Boldt P., Wirnitzer G., Leitzmann C., Tanous D., Motevalli M., Rosemann T., Knechtle B. (2022). Health status of recreational runners over 10-km up to ultra-marathon distance based on data of the NURMI Study Step 2. Sci. Rep..

[13] Waśkiewicz Z., Nikolaidis P.T., Gerasimuk D., Broysiuk Z., Rosemann T., Knechtle B. (2019). What motivates successful marathon runners? The role of sex, age, education, and training experience in Polish runners. Front. Psychol..

[14] Malchrowicz-Mòsko E., Poczta J. (2018). Running as a form of therapy socio-psychological functions of mass running events for men and women. Int. J. Environ. Res. Public Health.

[15] Allender S., Cowburn G., Foster C. (2006). Understanding participation in sport and physical activity among children and adults: A review of qualitative studies. Health Educ. Res..

[16] ASICS New Study Explores the World’s New-Found Love of Running. https://corp.asics.com/en/press/article/2020-06-09-1.

[17] Wirnitzer K.C. (2020). Vegan diet in sports and exercise—Health benefits and advantages to athletes and physically active people: A narrative review. Int. J. Sports Exerc. Med..

[18] American College of Sports Medicine Trending Topic/Nutrition. Indianapolis/USA..

[19] British Dietetic Association Adult Food Facts. https://www.bda.uk.com/food-health/foodfacts/adult-food-facts.html.

[20] Melina V., Craig W., Levin S. (2016). Position of the Academy of Nutrition and Dietetics: Vegetarian Diets. J. Acad. Nutr. Diet..

[21] Eker S., Garcia D., Valin H., Ruijven B. (2021). Using social media audience data to analyse the drivers of low-carbon diets. Environ. Res. Lett..

[22] Wirnitzer K.C., Grumezescu A.M., Holban A.M. (2018). Vegan nutrition: Latest boom in health and exercise. Therapeutic, Probiotic, and Unconventional Foods.

[23] Fiona Oakes Foundation (2014). World Records. https://www.fionaoakesfoundation.co.uk/world-records.

[24] Scott Jurek (2015). Born to Run. http://www.scottjurek.com/about-scott.

[25] Barnard N.D., Goldman D.M., Loomis J.F., Kahleova H., Levin S.M., Neabore S., Batts T.C. (2019). Plant-based diets for cardiovascular safety and performance in endurance sports. Nutrients.

[26] Alvero-Cruz J.R., Carnero E.A., García M.A.G., Alacid F., Correas-Gómez L., Rosemann T., Nikolaidis P.T., Knechtle B. (2020). Predictive performance models in long-distance runners: A narrative review. Int. J. Environ. Res. Public Health.

[27] Gordon D., Wightman S., Basevitch I., Johnstone J., Espejo-Sanchez C., Beckford C., Boal M., Scruton A., Ferrandino M., Merzbach V. (2017). Physiological and training characteristics of recreational marathon runners. J. Sports Med..

[28] O’Connor P. (2021). Pain during a marathon run: Prevalence and correlates in a cross-sectional study of 1251 recreational runners in 251 marathons. Front. Sports Act. Living.

[29] Tiller N.B., Elliott-Sale K.J., Knechtle B., Wilson P.B., Roberts J.D., Millet G.Y. (2021). Do sex differences in physiology confer a female advantage in ultra-endurance sport?. Sports Med..

[30] Cheuvront S.N., Carter R., Deruisseau K.C., Moffatt R.J. (2005). Running performance differences between men and women: An update. Sports Med..

[31] Wirnitzer K., Motevalli M., Tanous D.R., Gregori M., Wirnitzer G., Leitzmann C., Rosemann T., Knechtle B. (2021). Sex differences in supplement intake in recreational endurance runners—results from the NURMI Study (Step 2). Nutrients.

[32] Coast J.R., Blevins J.S., Wilson B.A. (2004). Do gender differences in running performance disappear with distance?. Can. J. Appl. Physiol..

[33] Motevalli M., Wagner K.-H., Leitzmann C., Tanous D., Wirnitzer G., Knechtle B., Wirnitzer K. (2022). Female endurance runners have a healthier diet than males—results from the NURMI Study (Step 2). Nutrients.

[34] Romaratezabala E., Castillo D., Raya-González J., Rodríguez-Negro J., Aritzeta I., Yanci J. (2020). Health and wellness status perception of half-marathon runners: Influence of age, sex, injury, and training with qualified staff. Int. J. Environ. Res. Public Health.

[35] Linton L., Valentin S. (2020). Running coaches and running group leaders’ engagement with, and beliefs and perceived barriers to prehabilitation and injury prevention strategies for runners. Phys. Ther. Sport.

[36] Knechtle B., Tanous D.R., Wirnitzer G., Leitzmann C., Rosemann T., Scheer V., Wirnitzer K. (2021). Training and racing behavior of recreational runners by race distance—results from the NURMI Study (Step 1). Front. Physiol..

[37] Wilson P. (2016). Nutrition behaviors, perceptions, and beliefs of recent marathon finishers. Phys. Sportsmed..

[38] Wirnitzer K., Motevalli M., Tanous D., Wirnitzer G., Leitzmann C., Pichler R., Rosemann T., Knechtle B. (2022). Who is running in the D-A-CH countries? an epidemiological approach of 2455 omnivorous, vegetarian, and vegan recreational runners—Results from the NURMI Study (Step 1). Nutrients.

[39] Mensink G.B., Barbosa C.L., Brettschneider A. (2016). Prevalence of Persons Following a Vegetarian Diet in Germany. J. Health Monit..

[40] Wirnitzer K., Motevalli M., Tanous D., Wirnitzer G., Leitzmann C., Wagner K.-H., Rosemann T., Knechtle B. (2021). Training and racing behaviors of omnivorous, vegetarian, and vegan endurance runners—Results from the NURMI Study (Step 1). Nutrients.

[41] Nebl J., Haufe S., Eigendorf J., Wasserfurth P., Tegtbur U., Hahn A. (2019). Exercise capacity of vegan, lacto-ovo-vegetarian, and omnivorous recreational runners. J. Int. Soc. Sports Nutr..

[42] Boldt P., Knechtle B., Nikolaidis P., Lechleitner C., Wirnitzer G., Leitzmann C., Rosemann T., Wirnitzer K. (2018). Quality of life of female and male vegetarian and vegan endurance runners compared to omnivores—Results from the NURMI study (step 2). J. Int. Soc. Sports Nutr..

[43] Wirnitzer K., Boldt P., Lechleitner C., Wirnitzer G., Leitzmann C., Rosemann T., Knechtle B. (2019). Health status of female and male vegetarian and vegan endurance runners compared to omnivores-results from the NURMI Study (Step 2). Nutrients.

[44] Boldt P., Knechtle B., Nikolaidis P.T., Lechleitner C., Wirnitzer G., Leitzmann C., Wirnitzer K. (2018). Sex differences in the health status of endurance runners: Results from the NURMI Study (Step 2). J. Strength Cond. Res..

[45] Damsted C., Parner E., Sørensen H., Malisoux L., Hulme A., Nielsen R.Ø. (2019). The association between changes in weekly running distance and running-related injury: Preparing for a half marathon. J. Orthop. Sports Phys. Ther..

[46] Tokudome S., Kuriki K., Yamada N., Ichikawa H., Miyata M., Shibata K., Hoshino H., Tsuge S., Tokudome M., Goto C. (2004). Anthropometric, lifestyle and biomarker assessment of Japanese nonprofessional ultra-marathon runners. J. Epidemiol..

[47] Wirnitzer K.C., Kornexl E. (2008). Exercise intensity during an 8–day mountain bike marathon race. Eur. J. Appl. Physiol..

[48] Wirnitzer K., Kornexl E. (2014). Energy and macronutrient intake of a female vegan cyclist during an 8-day mountain bike stage race. Bayl. Univ. Med. Cent. Proc..

[49] Wirnitzer K., Seyfart T., Leitzmann C., Keller M., Wirnitzer G., Lechleitner C., Rüst C.A., Rosemann T., Knechtle B. (2016). Prevalence in running events and running performance of endurance runners following a vegetarian or vegan diet compared to non-vegetarian endurance runners: The NURMI Study. Springerplus.

[50] Word Health Organization (WHO) A Healthy Lifestyle—WHO Recommendations. http://www.euro.who.int/en/health-topics/disease-prevention/nutrition/a-healthy-lifestyle/body-mass-index-bmi.

[51] Word Health Organization (WHO) THE GLOBAL HEALTH OBSERVATORY. Explore a World of Health Data. Noncommunicable Diseases: Risk Factors. http://www.who.int/gho/ncd/risk_factors/bmi_text/en/.

[52] Bangsbo J., Blackwell J., Boraxbekk C., Caserotti P., Dela H., Evans A.B., Jespersen A.P., Gliemann L., Kramer A.F., Lundbye-Jensen J. Copenhagen Consensus Statement 2019: Physical Activity and Ageing. https://bjsm.bmj.com/content/53/14/856.

[53] Desbrow B., Slater G., Cox G. (2020). Sports nutrition for the recreational athletes. Aust. J. Gen. Pract..

[54] Deldicque L., Francaux M. (2015). Recommendations for healthy nutrition in female endurance runners: An update. Front. Nutr..

[55] McDougall C., McDougall J. (2013). Plant-based diets are not nutritionally deficient. Perm. J..

[56] American Dietetic Association (1980). Position Paper on the vegetarian approach to eating. J. Am. Diet. Assoc..

[57] Spencer E.A., Applyby P.N., Davey G.K., Key T.J. (2003). Diet and body mass index in 38000 Epic-Oxford meat-eaters, fish-eaters, vegetarians and vegans. Int. J. Obes. Relat. Metab. Disord..

[58] Orlich M., Fraser G. (2014). Vegetarian diets in the Adventist Health Study 2: A review of initial published findings 1,2,3,4. Am. J. Clin. Nutr..

[59] Neufingerl N., Eilander A. (2021). Nutrient intake and status in adults consuming plant-based diets compared to meat-eaters: A systematic review. Nutrients.

[60] Bloomer R.J., Gunnels T.A., Schriefer J.M. (2015). Comparison of a restricted and unrestricted vegan diet plan with a restricted omnivorous diet plan on health-specific measures. Healthcare.

[61] Wirnitzer K., Motevalli M., Tanous D., Gregori M., Wirnitzer G., Leitzmann C., Rosemann T., Knechtle B. (2021). Supplement intake in half-marathon, (ultra-)marathon and 10-km runners—Results from the NURMI study (Step 2). J. Int. Soc. Sports Nutr..

[62] Wirnitzer K., Motevalli M., Tanous D.R., Gregori M., Wirnitzer G., Leitzmann C., Hill L., Rosemann R., Knechtle B. (2021). Supplement Intake in Recreational Vegan, Vegetarian, and Omnivorous Endurance Runners—Results from the NURMI Study (Step 2). Nutrients.

[63] van der Wall E.E. (2014). Long-distance running: Running for a long life?. Neth. Health J..

[64] Hargreaves S., Raposo A., Saraiva A., Zandonadi R. (2021). Vegetarian Diet: An overview through the perspective of quality of life domains. Int. J. Environ. Res. Public Health.

[65] Wirnitzer K. (2021). Nachhaltig gesund—Vegane Ernährung in Bewegung und Sport. Fachz. Beweg. Sport.

[66] Turner-McGrievy G.M., Moore W.J., Barr-Anderson D. (2016). The Interconnectedness of diet choice and distance running: Results of the Research Understanding the NutritioN of Endurance Runners (RUNNER) Study. Int. J. Sport Nutr. Exerc. Metab..

[67] Craig W.J., Mangels A.R., American Dietetic Association (2009). Position of the American Dietetic Association: Vegetarian diets. J. Am. Diet. Assoc..

[68] Bundesministerium für Ernährung und Landwirtschaft (BMEL). https://www.bmel.de/SharedDocs/Downloads/DE/Broschueren/ernaehrungsreport-2021.pdf?__blob=publicationFile&v=5.

[69] FMCG Gurus FMCG GURUS: Understanding the Growing Increase of Plant Based Diets. www.fmcggurus.com.

[70] Springmann M., Godfray H., Rayner M., Scarborough P. (2016). Analysis and valuation of the health and climate change cobenefits of dietary change. Proc. Natl. Acad. Sci. USA.

[71] Heydenreich J., Kayser B., Schutz Y., Melzer K. (2017). Total energy expenditure, energy intake, and body composition in endurance athletes across the training season: A systematic review. Sports Med. Open.

[72] Medawar E., Huhn S., Villringer A., Witte V. (2019). The effects of plant-based diets on the body and the brain: A systematic review. Transl. Psychiatry.

[73] Greger M.A. (2020). Whole food plant-based diet is effective for weight loss: The evidence. Am. J. Lifestyle Med..

[74] Sedeaud A., Marc A., Marck A., Dor F., Schipman J., Dorsey M., Haida A., Berthelot G., Toussaint J.F. (2014). BMI, a performance parameter for speed improvement. PLoS ONE.

[75] Grivetti L., Applegate E. (1997). From Olympia to Atlanta: A cultural-historical perspective on diet and athletic training. Am. Soc. Nutr. Sci..

[76] Swissveg: Veg-Umfrage. http://www.swissveg.ch/veg-umfrage.

[77] Leitzmann C. (2014). Vegetarian nutrition: Past, present, future. Am. J. Clin. Nutr..

[78] Bärebring L., Palmqvist M., Winkvist A., Augustin H. (2020). Gender differences in perceived food healthiness and food avoidance in a Swedish population-based survey: A cross sectional study. Nutr. J..

[79] Ek S. (2015). Gender differences in health information behaviour: A Finnish population-based survey. Health Promot. Int..

